# Interleukin-10 polymorphisms affect the key periodontal pathogens in Chinese periodontitis patients

**DOI:** 10.1038/s41598-018-26236-4

**Published:** 2018-06-13

**Authors:** Ying Geng, Lu Li, Xiaoqian Wang, Fanzhen He, Yi Zhou, Mifang Yang, Yan Xu

**Affiliations:** 1 Jiangsu Key Laboratory of Oral Diseases, Nanjing Medical University, Nanjing, Jiang Su China; 2 Department of Periodontics, Affiliated Hospital of Stomatology, Nanjing Medical University, Nanjing, Jiang Su China

## Abstract

Interleukin-10 (IL-10) polymorphisms have been shown to affect IL-10 production. This study investigated the influences of IL-10 polymorphisms on the susceptibility to chronic periodontitis (CP) and aggressive periodontitis (AP), and their possible role in the quantity of subgingival bacteria *Aggregatibacter Actinomycetemcomitans* and *Porphyromonas gingivalis*. 92 CP patients, 83 AP patients and 91 periodontal healthy controls were recruited. Serum IL-10 concentration was analyzed by enzyme-linked immunosorbent assay (ELISA). Gene polymorphisms were determined by multiplex SNaPshot technique. Bacteria were quantified by real-time polymerase chain reaction with TaqMan MGB probes. Taking into account age, gender and periodontal status, IL-10-592 AA, -819 TT and ATA/ATA genotype occurred more frequently in patients with CP than in healthy controls. In CP cases, higher quantity of subgingival *A*. *actinomycetemcomitans* and lower serum IL-10 levels could be detected in homozygous ATA/ATA carriers. These findings indicate that variants in IL-10 promoter gene were not only associated with predisposition to chronic periodontitis but also affected the subgingival number of *A. Actinomycetemcomitans* in a Chinese Han population.

## Introduction

Periodontitis, as a multifactorial chronic inflammatory disease, affects an extremely large percentage of the human population and leads to tooth loss. Generally it emerges either with the chronic or the aggressive form^1^. Periodontitis is predominantly a bacterial infection, initiated by microorganisms which could cause the defense of host immune system in the subgingival plaque, and it is also influenced by both genetic and acquired risk factors^2–4^. In particular, growing evidence suggests that individual genetic factors may have influence on the host’s immune response to pathogens^5^. Reports have focused on the relationship between genetic variations and microbial colonization^6,7^. There are several factors contribute to the differences in subgingival microbial colonization among persons. The reason, to some extent, is that the genetic variants play an important role in deciding which species are able to colonize the host^6^. Nibali *et al*. elucidated part of the mechanism of periodontal infect genomics as follows: once bacteria have colonized the periodontal tissues, in susceptible individuals they can not only proliferate but also trigger the immune-pathological reactions which could lead to tissue destruction. Furthermore, increased inflammatory response to plaque accumulation in subjects carrying specific genes may enhance the chance of overgrowth of particular components of the opportunistic microbiota (such as *A. actinomycetemcomitans*)^7^. Understanding of the molecular basis for these various responses could improve our knowledge of pathogenesis of infectious diseases such as periodontitis.

Cytokines act as messengers to initiate, mediate and control immune and inflammatory responses^8,9^. It’s well known that the interplay of pro- and anti- inflammatory cytokines plays a crucial role in the progression of periodontitis^10,11^. Cytokine gene polymorphisms often affect cytokine expression profile, and then may regulate susceptibility to infection in part by affect the colonization of the periodontopathic bacteria^12–22^.

Interleukin-10 (IL-10) is the most potent anti-inflammatory cytokine as it induces T cell anergy and downregulates the production of some pro-inflammatory cytokines^23,24^. IL-10 promoter region is highly polymorphic, and three single nucleotide polymorphisms (SNPs) at positions -1082(rs1800896), -819(rs1800871), and -592 (rs1800872) have been reported to be tightly correlated with altered IL-10 production *in vitro*^25–27^. The three SNPs are in linkage disequilibrium, resulting in three preference haplotypes: ATA, ACC and GCC. IL-10 promoter haplotype ATA has been associated with low production of IL-10, while GCC and ACC have been identified as risk factors for high and intermediate IL-10 production respectively^25,28^. AndIL-10 polymorphisms may modulate the host’s risk for periodontitis through various IL-10 levels. This mechanism could be proved by the evidence that the mice lacking IL-10 gene were highly susceptible to alveolar bone loss in comparison with the normal controls^29,30^, which could attribute to the attenuation of anti-inflammatory and increase of pro-inflammatory mediators. To date, just a limited number of studies have been performed to assess the association between the above SNPs and either chronic periodontitis (CP) or aggressive periodontitis (AP)^21,31–38^, however, no clear consensus has been reached. Two meta-analysis taking ethnicity and sample size into consideration suggest that −819 and −592 polymorphisms seem to be genetic risk factors for CP^39,40^.

On the basis of these clinical data, IL-10 polymorphisms may influence the composition of the subgingival bacteria by regulating IL-10 levels, and then be responsible for susceptibility to periodontitis. The current clinical study was performed to investigate the distribution of IL-10 polymorphisms in Chinese patients suffering from CP and AP compared with the healthy controls using the multiple logistic regression analysis. A further aim was to examine whether the IL-10 genetic variants could influence the subgingival bacterial counts of two key periodontal pathogens *Aggregatibacter actinomycetemcomitans* and *Porphyromonas gingivalis*.

## Subjects and Methods

### Study population and clinical investigations

This study was approved by the Ethical Committee of Stomatological Hospital affiliated to Nanjing Medical University, Nanjing, China. The purposes and procedures of the study were explained and informed consents were obtained from all recruits. The study was also performed in accordance with the declaration of Helsinki.

Patients and controls were recruited from the Stomatological Hospital affiliated to Nanjing Medical University. All subjects were Han Chinese population. Inclusion criteria comprised partially or fully dentate patients (at least 14 natural teeth, including 10 posterior teeth, excluding third molars), systemically healthy with no evidence of known systemic modifiers of periodontitis such as rheumatoid arthritis, diabetes, and osteoporosis. Subjects who met the following criteria were excluded from the study: (1) systemic modifiers of periodontal disease, as described above; (2) current pregnancy or lactation; (3) administration of antibiotics or anti-inflammatory drugs in the past six months; (4) current and former smokers (a person who smoked at least one cigarette per day was considered as a smoker); (5) received periodontal therapy in the past six months.

After collection of medical and dental histories, clinical parameters were recorded. Probing depth (PD), clinical attachment loss (CAL) and bleeding on probing (BOP) were examined at six sites per tooth with Florida probe (Florida Probe Corporation, Gainesville, Florida, USA). All assessments were measured by only one experienced clinician; a randomly chosen sample of 53(20%) subjects was re-measured by the same examiner one day later in order to establish the intra-examiner variance. The intra-examiner reproducibility for PD and CAL was assessed by *kappa* statistic, and the score of *kappa* was 0.89 and 0.87 respectively.

Patients were clinically diagnosed as generalized CP or generalized AP in accordance with the criteria established in 1999 at the World Workshop for a classification of Periodontal Diseases and Conditions, which was based on clinical, radiographic and historical findings^1^. CP patients were selected if they showed an attachment loss in at least 30% of the teeth with a minimum PD of 4 mm and lesions distributed on more than two teeth in each quadrant, CP often occurs in adults (but it may also affect younger patients), in addition, periodontal destruction is consistent with the amount of plaque present and other local factors. AP patients were included if they meet the following criteria: the disease occurred often before the age of 35 years (but it may also affect older people); history of rapid attachment loss and bone destruction as well as familial aggregation (if available); the presence of more than eight teeth with CAL ≥ 5 mm and PD ≥ 6 mm, and more than three affected teeth that were not first molars or incisors; the amounts of microbial deposits or subgingival calculus less than what would be expected for the amounts of periodontal destruction. No case that produced doubt in classification was included in the study. Periodontal healthy controls (PH) were included if they did not show any attachment loss, PD ≤ 3 mm and no history of periodontal disease. It is worth mentioning that CAL ≥ 3 mm as a result of traumatic tooth brushing, overhanging dental fillings, etc., was not considered as a case of periodontitis.

### Microbiological assessment of subgingival plaque samples

The subgingival plaque samples were obtained from the deepest site (excluding teeth with hopeless prognosis) in each quadrant before subgingival scaling and root planning were done, in addition, the CAL and PD of sampled sites were recorded, that is, the average of data in deepest site of each quadrant. The subgingival plaque samples were collected by inserting a sterile paper points for 30 s into the pocket after carefully removing the supragingival plaque, and then the samples were pooled from 4 sites per individual. Preparation of bacterial DNA was carried out using the QIAamp DNA Mini kit (Qiagen, Germany) according to the manufacturer’s instructions.

A real-time PCR assay was applied to achieve quantification of the pathogens, and the methods were described previously^41,42^. The oligonucleotide primers and probes, designed by Primer Express (version 2.0) software, are listed in Table 1. The primers and probes were based on *A. actinomycetemcomitans*- and *P. gingivalis*- specific conserved regions from lktA^43^ and 16S rRNA genes, respectively. Identification of conserved regions was done by multiple sequence alignment with ClustalW software based on the published sequences. Primers and probes were checked for possible cross-hybridization with bacterial genes using the database similarity search program BLAST. The fluorescent TaqManMGB probes were dually labeled with a reporter dye FAM attached to the 5′ end and a quencher dye MGB attached to the 3′ end. The primers used in real-time PCR were also used in conventional PCR, which were performed to confirm the specificities of the primers as well as to obtain the species-specific gene products. Consequently, the primers for *A. actinomycetemcomitans* and *P. gingivalis* demonstrated specific amplification products of each bacterial species and did not amplify DNA from other species containing *P. intermedia* and *T. forsythia*.

**Table 1:** Oligonucleotide primers and probes for real-time PCR.

Primers and probes	Sequence (5′–3′)	Production (bp)	Target
*A. actinomycetemcomins*			
Forward	TTGATCGTGCGAGAATGCTT		
Reverse	ATCGCCGTTATAACCAAATTTCTT		
Probe	FAM-AGGAATACTCGAAACGC-MGB	65	lktA
*P. gingivalis*			
Forward	TACCCATCGTCGCCTTGGT		
Reverse	CGGACTAAAACCGCATACACTTG		
Probe	FAM-ATTTATAGCTGTAAGATAGGC-MGB	126	16S rRNA

Quantitative PCR was carried out in duplicates in ABI PRISM 7300 sequence detection system (Applied Biosystems, USA) with the following cycle profile: 95 °C for 30 s followed by 40 cycles of 95 °C for 5 s and 60 °C for 31 s.

### Serum IL-10 level estimation

Venous blood samples were centrifuged at 1500 rpm for 10 min, and serum was then collected and kept in −70 °C conditions until tested. The level of IL-10 in the serum was measured by using ELISA kits (Invitrogen, USA) according to manufacturer’s instructions. The IL-10 level was obtained by comparison with the standard curve prepared. The sensitivity for IL-10 ELISA’s was 1 pg/mL, IL-10 level below the limit of the assay’s detectability was scored as 0.

### Genetic studies

For genetic investigations, fresh blood samples were collected in ethylenediaminetetraacetic acid (EDTA)–treated tubes and stored at −70 °C. Preparation of genomic DNA was carried out using a QIAamp DNA blood Mini kit (Qiagen, Germany) in accordance with the manufacturer’s manual.

Genomic regions containing the IL-10-592, -819 and -1082 SNPs were amplified by PCR using the following primers: -592, 5′-AAGAGGTGGAAACATGTGCC-3′ (forward) and 5′-TACCCAAGACTTCTCCTTGC-3′ (reverse); -819, 5′-ATGGTGTACAGTAGGGTGAG-3′ (forward) and 5′-TTTCCACCTCTTCAGCTGTC-3′ (reverse); -1082, 5′-AGAAGTCCTGATGTCACTGC-3′ (forward) and 5′-AAGTCAGGATTCCATGGAGG-3′ (reverse). The investigated SNPs were genotyped by a single-base primer extension assay using the SNaPshot™ Multiplex kit (Applied Biosystems, USA), according to the manufacturer’s instructions. The following primers were used: -592, 5′-TTTTTTCACATCCTGTGACCCCGCCTGT-3′; -819, 5′-TTACCCTTGTACAGGTGATGTAA-3′; -1082, 5′-CACTACTAAGGCTTCTTTGGGA-3′ (Table 1).

### Statistical Analysis

The SPSS 19.0 package was used for statistical analysis, values of *p* < 0.05 were considered significant. Continuous, normally distributed variables were reported as means ± standard deviations (SD). The genotype distributions in all groups were found to be in Hardy–Weinberg equilibrium. Association between IL-10 polymorphisms and CP/AP were analyzed by multiple logistic regression analysis. Age, gender, BOP, PD and CAL were entered in the analyses as covariates. We used Kruskal-Wallis ANOVA or a Mann-Whitney U test to analyze the impact of genetic variants on the number of subgingival bacteria and IL-10 serum levels, and a Dunn-Bonferroni test for post hoc comparisons (for values were not distributed normally), as appropriate, and by multiple linear regression analysis adjusted for age, gender, BOP, PD and CAL.

## Results

### Patient demographics and clinical characteristics

Demographic information and periodontal parameters of participants and sampled sites were listed in Table 2.

**Table 2:** Demographic and clinical characteristics of three groups.

Variable	Chronic periodontitis (n = 92)	Aggressive periodontitis (n = 83)	Periodontal healthy controls (n = 91)
Average age at diagnosis (years)	42 ± 7.8	28 ± 6.1	45 ± 9.8
Female (%)	56.6	41.3	52.8
Early tooth loss due to periodontitis among relatives (%)	41.3*	50.6*	17.6
No. of lost teeth (n)	2.0 ± 1.4*	0.9 ± 1.4*	0.1 ± 0.3
Bleeding on probing (BOP, %)	78.4 ± 9.0*	81.2 ± 12.0*	12.1 ± 6.3
Probing depth (PD, mm)	5.7 ± 0.9*	6.0 ± 0.9*	1.7 ± 0.2
Clinical attachment loss (CAL, mm)	6.4 ± 1.0*	6.7 ± 0.9*	0.2 ± 0.3
Teeth with CAL ≥ 6 mm (%)	60.3*	57.6*	0
PD (sampled site, mm)	6.8 ± 0.5*	7.2 ± 0.5*	1.9 ± 0.4
CAL (sampled site, mm)	7.0 ± 0.5*	7.3 ± 0.6*	0

*There were significant differences between chronic periodontitis or aggressive periodontitis and periodontal healthy controls (P < 0.05), but there was no significant difference between chronic periodontitis and aggressive periodontitis.

In comparison with the PH group, no statistically significant difference in gender could be detected. In accordance with the inclusion criteria, the AP patients are younger than CP and PH individuals, but there were no significant differences. Among their relatives, both patient groups reported significantly more often early tooth loss as a consequence of periodontitis (*p* < 0.001). Mean values of periodontal status, such as BOP (%), PD (mm) and CAL (mm) in patient groups were significantly higher than those in PH group. Severe generalized attachment loss for both patient groups was indicated via the results that the mean values for both PD (mm) and CAL (mm) were ≥ 5 mm, and the mean percentage of teeth with CAL ≥ 6 mm was more than 50%.

### Quantitative Detection of Periodontal pathogens

Sensitivity of the real-time PCR assay was evaluated using 10^0^ to 10^8^ plasmid copies of each pathogen. Limit for minimum detection was 10^2^ cells, and the sample was recognized as negative if its initial target genes were less than 10^2^.

**Figure 1:**
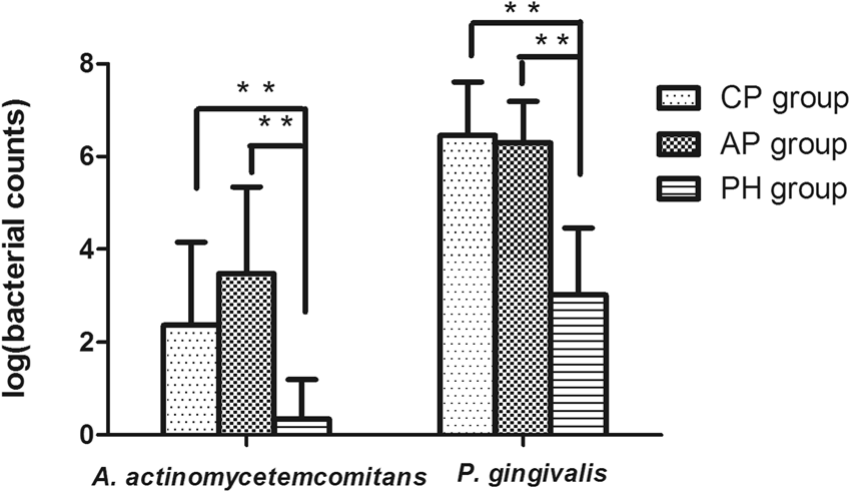
Bacterial counts (through logarithmic transformation) of *A. actinomycetemcomitans* and *P. gingivalis*. No significance (NS) for both pathogens by Mann-Whitney U test between CP and AP groups. Patients from CP and AP groups showed significant higher number of both pathogens compared with PH controls (P < 0.001 by Mann-Whitney U test). **Significant (p < 0.001 by Mann-Whitney U test) PH, periodontal healthy; CP, chronic periodontitis; AP, aggressive periodontitis.

When the bacterial counts were compared, there was a great variability between subjects (Fig. 1). Evaluated from the mean value, no significant difference was detected in the number of *A. actinomycetemcomitans* and *P. gingivalis* between CP and AP groups. The difference of the two bacterial load was analyzed within each group, and the amount of *P. gingivalis* was much higher in comparison with that of *A. actinomycetemcomitans* (*p* < 0.001). Further, periodontal patients showed a higher number of the tested pathogens, compared to healthy controls (*p* < 0.001).

### Allele and genotype distribution of three IL-10 SNPs

Association between IL-10 polymorphisms and CP/AP were analyzed by multiple logistic regression analysis. Age, gender, BOP, PD and CAL were entered in the analysis as covariates.

The A allele and AA genotype at position -592 (*p* = 0.03, OR = 2.32, 95%CI = 1.45–3.41; *p* = 0.023, OR = 4.23, 95%CI = 1.26–6.78), and the T allele and TT genotype at position -819 (*p* = 0.034, OR = 1.88, 95%CI = 1.32–2.59; *p* = 0.021, OR = 3.59, 95%CI = 1.45–7.06) occurred more frequently in patients with CP than in healthy controls. Related with AP, no association between IL-10 polymorphisms and AP was found (Table 3). According to the haplotype analysis, the dominant haplotype was ATA (-1082-819-592) in three groups (57.7% in PH group, 70.1% in CP group, and 64.5% in AP group). There was a trend that the haplotype ATA was expressed more in patients with CP (*p* = 0.076, OR = 1.72, 95%CI = 0.93–2.65) (Table 4). In patients with CP, the frequency of the combination ATA/ATA was increased compared with healthy controls (*p* = 0.017, OR = 2.43, 95%CI = 1.21–3.83) (Table 4).

**Table 3:** Genotype and allele frequencies of SNPs in IL-10 promoter gene of three groups and results of logistic regression analyses.

Single nucleotide	PH	CP	AP	CP vs.PH		AP vs.PH	
polymorphism	n (%)	n (%)	n (%)	p	OR (95%CI)	p	OR (95%CI)
IL-10-592 Genotype	n = 91	n = 92	n = 83				
CC	17 (18.7)	7 (7.6)	10 (12)		1		1
AC	42 (46.2)	39 (42.4)	36 (43.4)	0.146	2.45 (0.93–5.44)	0.341	1.69 (0.44–3.52)
AA	32 (35.2)	46 (50)	37 (44.6)	0.023	4.23 (1.26–6.78)	0.163	2.14 (0.71–5.63)
A carrier	106 (58.2)	131 (71.2)	110 (66.3)	0.03	2.32 (1.45–3.41)	0.198	1.12 (0.73–1.85)
IL-10–819 Genotype	n = 91	n = 92	n = 83				
CC	17 (18.7)	7 (7.6)	10 (12)		1		1
TC	39 (42.9)	40 (43.5)	38 (45.8)	0.205	1.25 (0.67–2.34)	0.361	1.52 (0.47–2.65)
TT	35 (38.5)	45 (48.9)	35 (42.2)	0.021	3.59 (1.45–7.06)	0.322	1.96 (0.45–5.67)
T carrier	109 (59.9)	130 (70.7)	108 (65.1)	0.034	1.88 (1.32–2.59)	0.184	1.36 (0.79–2.13)
IL-10-1082 Genotype	n = 91	n = 92	n = 83				
GG	2 (2.2)	1 (1.1)	1 (1.2)		1		1
AG	16 (17.6)	9 (9.8)	10 (12.0)	0.879	1.02 (0.83–11.79)	0.933	1.12 (0.57–12.47)
AA	73 (80.2)	82 (89.1)	72 (86.7)	0.654	2.67 (0.65–24.29)	0.704	2.87 (0.2–24.24)
A carrier	162 (89.0)	173 (94.0)	154 (92.8)	0.584	2.31 (0.75–7.63)	0.653	1.92 (0.55–5.28)

PH, periodontal healthy subjects; CP, chronic periodontitis subjects; AP, aggressive periodontitis subjects.

**Table 4:** Distribution of IL-10 haplotypes (arranged as allele frequencies) and IL-10 combinations (arranged as genotype frequencies) of three groups and results of logistic regression analyses.

Haplotype	PH (n = 182)	CP (n = 184)	AP (n = 166)	CP vs.PH		AP vs.PH	
-1082-819-592	n (%)	n (%)	n (%)	p	OR (95%CI)	p	OR (95%CI)
ATA	105 (57.7)	129 (70.1)	107 (64.5)	0.076	1.72 (0.93–2.65)	0.105	1.27 (0.78–2.31)
Others	77 (42.3)	55 (29.9)	59 (35.5)				
ACC	52 (28.6)	41 (22.3)	43 (25.9)	0.745	0.84 (0.57–1.42)	0.886	1.04 (0.58–1.80)
Others	130 (71.4)	143 (77.7)	123 (74.1)				
Genotype	PH (n = 91)	CP (n = 92)	AP (n = 83)	CP vs.PH		AP vs.PH	
-1082-819-592/-1082-819-592	n (%)	n (%)	n (%)	p	OR (95%CI)	p	OR (95%CI)
ATA/ATA	31 (34.1)	44 (47.8)	34 (41.0)	0.017	2.43 (1.21–3.83)	0.149	1.57 (0.84–2.60)
others	60 (65.9)	48 (52.2)	49 (59.0)				
ATA/ACC	28 (30.8)	30 (32.6)	31 (37.3)	0.553	1.32 (0.42–2.75)	0.447	1.61 (0.77–2.87)
others	63 (69.2)	62 (67.4)	52 (62.7)				

PH, periodontal healthy subjects; CP, chronic periodontitis subjects; AP, aggressive periodontitis subjects.

### The impact of genetic variants on the number of subgingival bacteria and the serum IL-10 level

**Figure 2:**
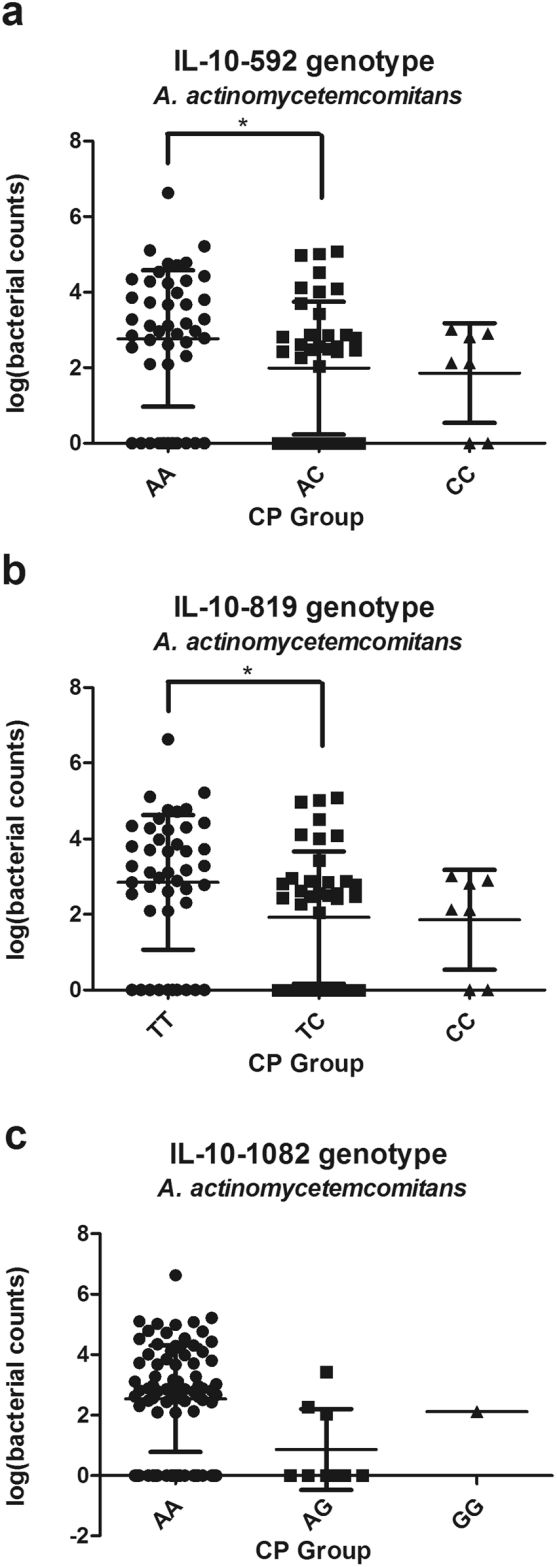
Association between SNPs in IL-10 promoter gene (IL-10-592, -819 and -1082 genotype) and the subgingival number of *A. actinomycetemcomitans* in CP group. *Significant (p < 0.05 by Kruskal-Wallis ANOVA test followed by Dunn-Bonferroni test) CP, chronic periodontitis.

**Figure 3:**
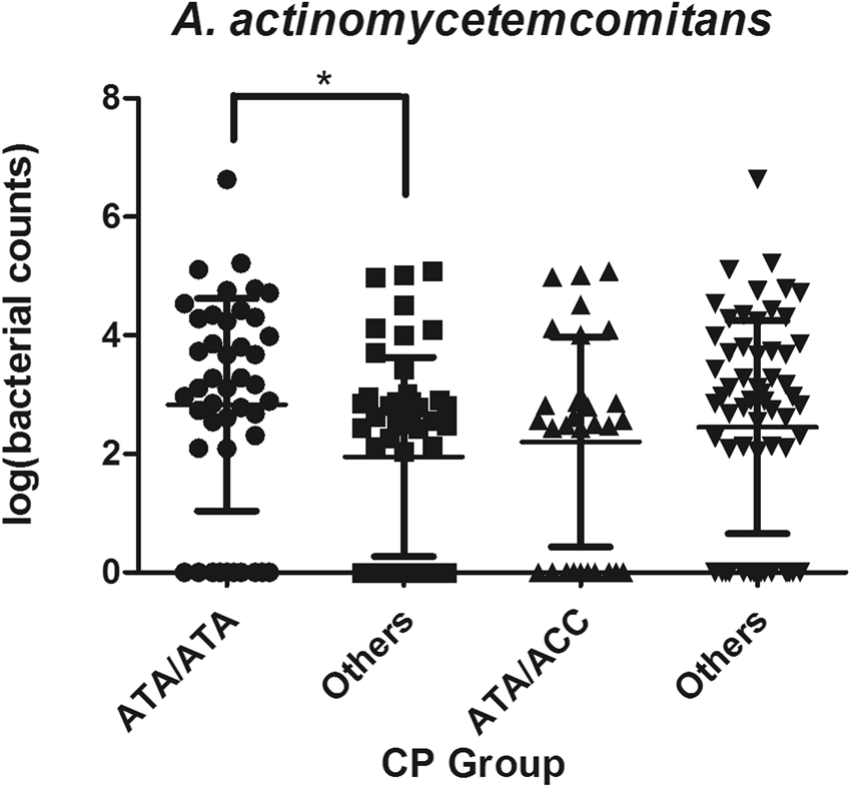
Association between the IL-10 haplotype and the subgingival number of *A. actinomycetemcomitans* in CP group. *Significant (p < 0.05 by Mann-Whitney U test) CP, chronic periodontitis.

The amount of *A. actinomycetemcomitans* was statistically different among -592 AA, AC and CC groups in patients with CP (χ^2^ = 9.29 *p* = 0.028), and AA individuals showed higher bacterial counts of *A. actinomycetemcomitans* than AC subjects in the following all pairwise multiple comparison test (*p* = 0.035). The amount of *A. actinomycetemcomitans* also differed among -819 TT, TC and CC groups in patients with CP (χ^2^ = 10.13 *p* = 0.017), and TT individuals showed higher bacterial counts of *A. actinomycetemcomitans* than TC subjects in post hoc comparisons (*p* = 0.023) (Fig. 2). Moreover, the amount of subgingival *A. actinomycetemcomitans* was significantly increased in ATA/ATA-positive individuals of CP subjects (*p* = 0.009) (Fig. 3). It seemed that IL-10 polymorphisms may not influence the amount of *A. actinomycetemcomitans* in AP cases and the healthy controls (data were not shown). Moreover, no significant association between IL-10 polymorphisms and the bacterial load of *P. gingivalis* was observed in any group (data were not shown). Further multiple regression analysis using the stepwise method was carried out, ATA/ATA carriers were associated with increased bacterial counts of *A. actinomycetemcomitans* in CP group after adjustment for age, gender, BOP, PD and CAL (*p* = 0.013) (Table 6).

Serum IL-10 levels differed among -592 AA, AC and CC groups in CP subjects (χ^2^ = 8.74 *p* = 0.028), lower IL-10 levels were significantly detected in subjects carrying the AA genotype compared with AC genotype (*p* = 0.036). Serum IL-10 levels were also statistically different among -819 TT, TC and CC groups in CP subjects (χ^2^ = 8.32 *p* = 0.031), and TT subjects presented significant lower IL-10 levels than TC subjects in post hoc comparisons (*p* = 0.04). Serum IL-10 concentrations was significantly lower in ATA/ATA-positive individuals of CP subjects (*p* = 0.033) (Table 5). However, any of the IL-10 haplotypes had no significant influence on the serum IL-10 levels in the healthy group, the AP group and the whole diseased group (data were not presented). Moreover, multiple linear regression analysis by stepwise method, adjusted for age, gender, BOP, PD and CAL, was used to investigate the impact of the above IL-10 haplotypes in subjects with CP. The analysis revealed that ATA/ATA-positive individuals were still related to lower serum IL-10 levels among CP patients (*p* = 0.021) (Table 6).

**Table 5:** Serum level (mean ± SD) of IL-10 in relation to genetic polymorphisms in CP group.

polymorphism	genotype	Serum IL-10 (pg/ml)
IL-10-592	CC (n = 7)	6.2 ± 5.5
	AC (n = 39)	7.6 ± 7.4
	AA (n = 46)	4.8 ± 4.1*
IL-10-819	CC (n = 7)	6.2 ± 5.5
	TC (n = 40)	7.6 ± 7.3
	TT (n = 45)	4.8 ± 4.1*
1082-819-592/1082-819-592	ATA/ATA (n = 44)	4.8 ± 4.0^§^
	Others (n = 48)	7.2 ± 5.8

CP, chronic periodontitis.

*Significant (p < 0.05 by Kruskal-Wallis ANOVA test followed by Dunn-Bonferroni test) IL-10-592 AA subjects vs. AC subjects; IL-10-819 TT subjects vs. TC subjects.

^§^Significant (p < 0.05 by Mann-Whitney U test).

**Table 6:** Results of multiple regression analyses for the impact of genetic variants on the number of subgingival bacteria and the serum IL-10 level.

	Unstandardized coefficients		Standardized coefficients	T	P
	B	Standard error	β		
Subgingival counts of *Aa*					
ATA/ATA	0.79	0.46	0.25	2.43	0.013
Serum level of IL-10					
ATA/ATA	−2.03	1.58	−0.96	−3.21	0.021

*Aa, A. actinomycetemcomitans*.

## Discussion

The present study analyzed the distribution of three SNPs of IL-10 promoter gene among patients with CP, patients with AP and periodontal healthy controls in Chinese Han population. Furthermore, possible impact of IL-10 genetic variants on the quantity of subgingival bacteria *A. actinomycetemcomitans* and *P. gingivalis* were investigated.

The microbial analysis revealed that the two bacteria investigated were also found in the healthy controls, however, with significantly lower number compared to the patient groups (Fig. 1). It suggests that besides periodontal pathogens, the host immune response and its genetic regulation and control may be crucial to the development of periodontitis. Moreover, the amount of *A. actinomycetemcomitans* cannot be used to distinguish patients with AP and patients with CP due to its great variability between individuals within groups (Fig. 1), which was in accordance with some recent studies^44,45^.

All the periodontitis patients involved expressed severe generalized attachment loss, hence, they were suitable for the identification of genetic risk factors. Multiple logistic regressions revealed that that IL-10-592 AA, -819 TT and ATA/ATA genotype may confer a slight increase in the risk for CP after adjustment for age, gender and periodontal status (Tables 3, 4). With regard to the -592 SNP, the related studies most presented the similar trends in their populations, including two meta-analysis^32,33,35,39,40,46^. In agreement with our results concerning the -819 SNP, only one study suggested that -819 CC genotype may decrease the risk for CP^33^, while the others yielded negative associations. As to the -1082 loci, negative result was in accordance with previous studies. It was showed that in Brazilian female cases, there was a trend with the predominance of haplotype ATA in CP group (p = 0.061)^33^. In German subjects the haplotype ATA could increase the odds ratio for AP^21^. In Taiwanese population the ATA/ATA haplotype could increase the risk of AP and individuals with the ATA/ACC genotype were less susceptible to CP^35^. A possible reason for these conflicting results might be based on ethnic differences in the distribution of IL-10 polymorphisms. The genotype frequencies of IL-10 SNPs in Chinese subjects vary from those of Caucasians^47^, but they are racially close to those of Japanese^48^ and Taiwanese individuals^35^. The ATA haplotype was dominant in Asian population, and the frequency of it was 0.64 in our study. GCC and ACC haplotypes were in the majority among Caucasians whereas ATA just accounted for 0.21. In addition, other factors, such as the sample size, oral hygiene and smoking can have impact on the inconsistent genetic results.

The positive associations between IL-10 certain genotypes and CP might be explained by the following reasons. IL-10 promoter haplotype ATA has been related to low production of IL-10^25^. After the initial colonization of the pathogens in the periodontal region of IL-10 hypo-producers, reduced IL-10 levels might modulate the host immune response, with attenuation of anti-inflammatory and increase of pro-inflammatory mediators^49^. For instance, it might result in increased production of tumor necrosis factor- α, which was known for its important role in alveolar bone loss^50^. Moreover, the absence of IL-10 could result in accelerated alveolar bone loss^29,30^. Obviously, individuals with certain genotypes were more likely to develop CP characterized by alveolar bone loss.

In the present study, we obtained a positive association between IL-10 ATA/ATA genotype and lower serum IL-10 levels, in addition, a positive relation to increased subgingival bacterial counts of *A. actinomycetemcomitans* by multiple regression analysis in Chinese patients with chronic periodontitis for the first time (Table 6). The findings indicated the possible effect of IL-10 polymorphisms on the periodontopathic bacteria. This study supported the hypothesis that the host genotype can influence the composition of the subgingival microbiota^7^. However, limiting the microbial analysis to two periodontal pathogens may overlook the possible influence on subgingival microbiota, so comprehensive microbiological assessment should be adopted in the following study. Moreover, oral hygiene may also influence the subgingival colonization^51,52^, based on this point, plaque index should also be listed as a covariate in the multiple regression analysis, but we lack these data, so more comprehensive data should be collected when the participants are recruited in further study. Hyper-inflammatory genotypes (IL-10 ATA/ATA) cluster presented higher subgingival counts of *A. actinomycetemcomitans* and higher risk for chronic periodontitis which revealed that complex interactions between the host genetic variants and the subgingival microbiota are at the basis of predisposition to periodontitis. The reasons for the above associations are not entirely clear, we hypothesize a possible explanation for the findings. We assume that ATA/ATA carriers (IL-10 hypo-producers) may be prone to the growth of bacteria, such as *A. actinomycetemcomitans*. After the initial colonization of the pathogens in the periodontal region of IL-10 hypo-producers, they may benefit from the hyperactivation of host cells, the stimulation of the inflammatory cascade and the increased production of multiple inflammatory cytokines^49,53^ due to the effect of IL-10 genetic variants. In a word, the pathogens could gain more favorable environment for their survival and well overgrowth in inflamed areas in subjects with specific genotype. And increased pathogens in turn may affect the local tissues which would lead to enhanced inflammation and periodontal pocket formation. In addition, the absence of IL-10 could result in accelerated alveolar bone loss^29,30^. Increased alveolar bone loss and deepened periodontal pockets would further favor the growth of *A. actinomycetemcomitans* due to the anaerobic environment. The study revealed that IL-10 ATA/ATA individuals (with lower serum IL-10 levels) were more susceptible to CP, and it may be partly caused by higher subgingival counts of *A. actinomycetemcomitans* which increased the risk for characteristic periodontal tissue destruction. In the present study, there was no association between IL-10 polymorphisms and AP. Moreover, any of the IL-10 haplotypes had no significant influence on the serum IL-10 levels and the amount of *A. actinomycetemcomitans* and/or *P. gingivalis* in the AP group. The above results may be related to the sample size, the exclusion of current and former smokers, and the lack of plaque index. However, the results may indicate that the susceptibility to AP might be not closely tied with the change of *A. actinomycetemcomitans* and/or *P. gingivalis* caused by IL-10 genetic variants to some extent.

To date several clinical studies have been conducted to assess the influence of some cytokine genes polymorphisms on the subgingival microbiota. Among these, IL-6^12,13,15^ and IL-1^16–18,22^ polymorphisms have received most attention, and it has been proved that they were associated with subgingival detection of *A. actinomycetemcomitans* and *P. gingivalis*. IFN-γ-AA carriers had a lower odds ratio for the presence of *A. actinomycetemcomitans*^54^. IL-2-330,166 TT: TT combination seemed to affect the occurrence of *P. gingivalis* and bacteria of the ‘red’ complex^19^. The Q551R IL-4R polymorphism was associated with the presence of T. forsythia^20^. In addition, a study obtained IL-10 ATA haplotype was associated with increased odds ratio for AP among Caucasians population and the presence of *P. intermedia* was found to be decreased in ATA- positive individuals^21^. The results are contradictory to ours, and the possible reasons for these conflicting results might be based on ethnic differences in the distribution of IL-10 polymorphisms, sample size, or sensitivity of molecular assessment for periodontopathic bacteria.

In conclusion, despite of the limitations of sample size, the results of this study suggest that the possible influence of IL-10 polymorphisms on the susceptibility to chronic periodontitis. In addition, IL-10 ATA/ATA genotype is associated with the subgingival quantity of *A. actinomycetemcomitansi* in subjects with chronic periodontitis. This study supports the hypothesis that complex interactions between the host genetic variants and the subgingival microbiota are at the basis of susceptibility to periodontitis.
